# Protecting our digital medicine infrastructure

**DOI:** 10.1038/s41746-019-0177-y

**Published:** 2019-10-02

**Authors:** Ted Adams, Matthew Connor, Robyn Whittaker

**Affiliations:** 1 Southport and Ormskirk NHS Trust, Wigan Road, Ormskirk, Lancashire L39 2AZ UK; 2 St Helens and Knowsley Health Informatics Service, Pavilion Building, Alexandra Business Park, St. Helens, WA10 3TP UK; 3 Health Informatics & Technology, School of Population Health, The University of Auckland, Auckland, 1142 New Zealand

**Keywords:** Health policy, Health services, Policy

On Friday 12th May at ~11:30 a.m., the information technology (IT) helpdesk at our hospital received four calls from people around our organisation, who reported seeing a strange message appear on their screen. The message displayed would become familiar to many thousands of computer users over the ensuing few hours and days, and to millions more beyond that as the extent and fall out of the *Wannacry* cyberattack became clearer.

At our hospital, we had taken cyber security seriously even before the attack, and had deployed a protective Microsoft patch to over 1500 desktop computers in the weeks before the *Wannacry* cyberattack. This, along with our immediate response to the threat—all trust systems were shut down within 45 min of the initial call—meant that only 0.6% of our hardware was infected. Our organisation declared the cyberattack a major incident, which was later shown to be the correct response, given the way the organisation would be forced to handle the situation over the next week or so. It was quickly clear, however, that the standard major incident response to a cyberattack needed some modification because of the type of threat we were confronted with. This incident was no motorway pileup 10 miles away leading to a great influx of patients; instead this event would threaten the internal workings of our entire organisation.

The response to the cyberattack affected all computer systems including desktops and servers. The internal networks were shut down, and local links to networks and links to the internet were severed. We were forced to rely on telephone calls (initially mobile calling only), radio handsets and paper for all communication. Over the ensuing 7 days, the systems were gradually brought back on line with clinically critical systems at the top of the priority list.

In their inaugural editorial, the Editors-in-Chief Steve Steinhubl and Eric Topol^1^ hoped that the need for the journal’s existence would eventually diminish as digital medicine became “just plain medicine.” The *Wannacry* attack exposed how much digital medicine and digital systems have spread into our healthcare organisations and the entire NHS. Not only was the electronic patient notes system brought down, the response to the attack also shut down seemingly unrelated areas like the catering system and the finance system, which took varying amounts of time to restart. The efficiency with which digital systems allow healthcare organisations to function is often underestimated—potentially because we never turn them all off at the same time. The tendrils of digital systems stretch into every corner of an organisation, from parking your car at the hospital to getting your medication from the pharmacy. Unfortunately, each of these digital systems can be very specific to individual departments and are critical to their safe functioning, yet knowledge and responsibility of the systems within departments can vary greatly. IT has become an integral component of all organisations, possibly without all departments being aware of how dependent they have become on their digital systems.

The paper by Ghafur et al. ^2^ demonstrates that a cost can be ascribed to the failure of these digital systems, and that cost is considerable. Certainly, the conclusions from their study reflect our own experience in which considerable effort was put into keeping front line services open and safe, while elective work was sacrificed. This was the primary consequence of losing staff time to the inevitable, inefficient, but safe ways of working that were put in place while systems were down.

When the benefits of digital medicine are discussed, they are often discussed in the context of artificial intelligence, big data, and improved access to care for patients. The focus of the discussion is not about the barriers at the entrance to the hospital car park. We are not suggesting that the grander benefits of digital medicine are not possible nor desirable, just that they are dependent on a functioning supportive operational digital infrastructure. The digital medicine infrastructure is essential to the safe operation of healthcare, and excellent cyber security protection is needed now.

Experts in cyber security agree that a future healthcare cyberattack will happen. Specific actions around preparedness and prevention for the UK were discussed in the NHS’s response to the attack.^3^ This response cites the importance of the training and development of staff to reduce the impact of the next attack. Currently, cyber security is nested within the larger remit of information governance. For information and technology professionals, this may seem like the obvious home for cyber security. Indeed, placing an organisation’s corporate leadership and responsibility for information protection and cyber security together makes sense. However, organisations may need the average healthcare professional’s eyes, ears, and mouse fingers to be more attuned to the cyber security threats than perhaps they are now. An organisation’s stability will be enhanced by cyber security awareness, but also by having robust clinical digital leaders who understand and can explain the current benefits and limitations of digital medicine that exists within their organisational boundary.

That said, staff working in healthcare *are* aware of the dangers posed by cyber criminals. When the *Wannacry* outbreak began at our organisation, it was the calls from our staff to the IT helpdesk that got our initial attention. These calls were then confirmed and backed up by problems on our network, leading to an immediate professional response. It is imperative that we make it as easy as possible for staff to recognise what could be an attack, how and when to report an attack, and for IT professionals to act on those reports without delay. We must be willing to do whatever is necessary to protect our digital medicine infrastructure from future attacks because it is not a question of if an attack will happen but a question of when and where will it happen next.
